# Literary Notes

**Published:** 1902-11

**Authors:** 


### LITERARY NOTES.

THE *Hot Springs Medical Journal* appeared in September in a new cover which adds much to the appearance of this smart magazine.

---

THE *Journal of the Michigan State Medical Society* is the name of a journal published in Detroit, Mich., which made its first appearance in September. It is announced as the official organ of the state and county societies of Michigan and is published monthly under the supervision of the council of the state society. The appearance of the first number is such as to give promise of the success of the magazine.

THE *Medico-Legal Bulletin* presented itself in October as the first journal in the world devoted strictly to the interests of the medical profession from a legal viewpoint. It is published monthly by the Physicians' Defense Company, Fort Wayne, Indiana.

